# Preparation and Comprehensive Performance Evaluation of Hydrophobic Anti-Icing Coating Materials for Highway Pavements

**DOI:** 10.3390/ma18204778

**Published:** 2025-10-19

**Authors:** Xin Xu, Yingci Zhao, Qi Wang, Mingzhi Sun, Yuchun Li

**Affiliations:** 1 Research Institute of Highway Ministry of Transport, Beijing 100088, Chinayc.li@rioh.cn (Y.L.); 2 Chongqing Fengjian Expressway Co., Ltd., Chongqing 404600, China; 3 National Observation and Research Station of Corrosion of Road Materials and Engineering Safety in Dadushe Beijing, Beijing 100088, China

**Keywords:** cement pavement, asphalt pavement, hydrophobic anti-icing coating, silane oligomers, comprehensive pavement performance

## Abstract

In winter, some roads face the problems of severe rain accumulation and ice formation, which pose major risks to traffic safety and result in substantial economic losses. With the development of hydrophobic materials, hydrophobic coatings have gradually gained attention as a novel anti-icing technology. In this study, utilizing vinyl triethoxysilane (VTES) as the monomer and benzoyl peroxide (BPO) as the initiator, a hydrophobic anti-icing coating for highway pavements was prepared through the free radical polymerization method. Through designing the icing rate test and ice–pavement interface adhesion strength test, combining the contact angle test technology, wet wheel abrasion test, and pendulum friction coefficient test, the anti-icing performance, durability, and skid resistance performance of the hydrophobic anti-icing coating under the three types of mixtures of asphalt concrete (AC-13), Portland cement concrete (PCC), and porous asphalt concrete (PAC-13) were evaluated. The results indicate that when the surface layer of the pavement was sprayed with anti-icing coating, the water was dispersed in a semi-spherical shape and easily rolled off the road surface. Compared to uncoated substrates, the anti-icing coating reduced the icing rate on the surface by approximately 25%. Comparing with the uncoated pavements mixtures, for AC-13, PCC, and PAC-13 pavements, the ice–pavement interface adhesion strength after the application of hydrophobic anti-icing coating reduced by 30%, 79% and 34%, respectively. Both cement pavements and asphalt pavements, after the application of hydrophobic anti-icing coating, expressed hydrophobic properties (contact angle of 131.3° and 107.6°, respectively). After wet wheel abrasion tests, the skid resistance performance of pavement surfaces coated with the hydrophobic anti-icing coating met the specification requirements. This study has great significance for the promotion and application of hydrophobic anti-icing technology on highway pavements.

## 1. Introduction

In winter, some roads face the problems of severe rain accumulation and ice formation, which pose major risks to traffic safety and result in substantial economic losses [1]. At present, there are two main methods of road anti-icing and snow melting, which are active methods and passive methods. Active methods of anti-icing are to endow the pavement with snow and ice melting functions by designing pavement structures, modifying paving material properties, and incorporating functional facilities. Passive methods mainly include manual removal, mechanical techniques, and the spreading of de-icing salts [2,3]. Among them, the spreading of de-icing salts is the predominant measure of a passive method (e.g., chlorine salts) [4,5]. However, under an environment of cooler temperatures and substantial ice accumulation, the de-icing salts may difficult to melt snow and ice rapidly and effectively. Moreover, the excessive use of de-icing salts can also lead to the deterioration of pavement materials and structures, as well as environmental pollution, including soil and water contamination. Existing active de-icing technologies are hardly able to meet the requirements of low cost, ease of construction, and high de-icing performance simultaneously. Therefore, developing a de-icing method with the characteristics of high efficiency, environmental friendliness, convenient construction, and low cost has become a focal point in road engineering [6].

Hydrophobic coatings have received increasing attention in recent years due to their hydrophobic and anti-icing properties. Hydrophobic materials can effectively reduce the amount of ice formation and delay the time of ice crystal formation. Thus, water cannot accumulate abundantly on the surface of the substrate. This characteristic of hydrophobic materials can fundamentally solve the problem of icing formation on the surface of the substrate. In recent years, hydrophobic materials have been extensively studied in various fields and employed to develop smart materials for practical applications, including self-cleaning, anti-corrosion, anti-fouling, biomedical engineering, anti-icing, and oil–water separation [7,8,9,10,11]. Particularly in the applications of anti-icing and de-icing in the fields of aerospace, power transmission lines [12,13]. In addition, it has the advantages of convenient construction and low cost [14,15]. However, the application of hydrophobic materials in the field of road anti-icing is still at the stage of preliminary exploration.

Mobarakeh et al. [16] deposited a superhydrophobic thin film on an aluminum oxide substrate by low-pressure plasma polymerization of hexamethyldisiloxane. And the contact angle (CA) on the elaborated coating was shown to be 158° with a sliding angle of about 8°, and the superhydrophobic film showed ice-adhesion strength 3.5 times smaller than that of the untreated aluminum surface. Wang et al. [17] prepared a nanocomposite coating with hybrid nanofiller reinforcement and modified polymeric resin, exhibiting chemically and mechanically stable hydrophobic properties. By introducing polydimethylsiloxane to modify the epoxy resin, the hydrophobicity was improved, increasing the CA from 50° to 110°. However, in practical applications, the water resistance of the coating tends to decline rapidly due to friction or aging. The mechanical durability of the coating remains a key issue limiting its widespread adoption, especially on road surfaces, where wear is particularly prominent [18]. He et al. [19] developed a superhydrophobic anti-icing coating for asphalt surfaces. The coating was fabricated by synthesizing nano-SiO_2_-coated carbon nanotubes (CNTs) via sol–gel technology, followed by surface modification through dodecyltrimethoxysilane to obtain superhydrophobic nano-fillers, achieving a CA of 154.7°. However, after 200 sandpaper abrasion cycles, the coating exhibited an 11.4° reduction in CA. Xu et al. [20] developed a photothermal and electrothermal superhydrophobic coating by incorporating FAS-modified graphene (F-GPE) into epoxy resin as a filler for anti/de-icing under cold conditions. The coating achieved a CA close to 160°. After undergoing sandpaper abrasion and sand impact tests, the CA of the coating decreased by approximately 10°. Therefore, especially on road surfaces, hydrophobic coatings are subjected to multiple factors such as wheel load and temperature variations, which impose higher demands on their durability.

From the overview of the literature described above, it is worth noting that the resin-based coating materials can achieve satisfactory hydrophobic effects. However, when application in the road de-icing environment, it exhibits poor durability. In contrast, silane-based materials can penetrate deep into concrete pores through capillary action. And the chemical bonding between the silane-based materials and substrates can provide the superior stability for hydrophobic layers [21]. However, silane-based hydrophobic materials have mostly been used in the field of concrete anti-corrosion [22], and there are few research and practical applications in road surface de-icing.

Therefore, the objective of this study is to prepare a hydrophobic anti-icing material with silane oligomers as the main component, and to evaluate the comprehensive performance under the application on highway pavement. In this paper, the vinyl triethoxysilane (VTES) as the monomer and the benzoyl peroxide (BPO) as the initiator, a hydrophobic anti-icing coating for highway pavements has been synthesized by the method of free radical polymerization. Through designing the icing rate test and ice–pavement interface adhesion strength test, combining the CA test technology, wet wheel abrasion test, and pendulum friction coefficient test, the anti-icing performance, durability, and skid resistance performance of the hydrophobic anti-icing coating under the three road surface layer structures of AC-13, PCC, and PAC-13 were evaluated. This research holds significant importance for the application of hydrophobic materials in the field of road surface de-icing, and it also plays a positive role in promoting the development of active de-icing technologies on roads.

## 2. Experimental Method

### 2.1. Preparation of the Hydrophobic Coating

In this study, the BPO, as the initiator, and VTES, as the unsaturated organosilicon monomer, undergo a polymerization reaction by opening double bonds to form silane oligomers. And the silane oligomers were also the main components of the hydrophobic anti-icing coating for highway pavements in this paper.

**Figure 1:**
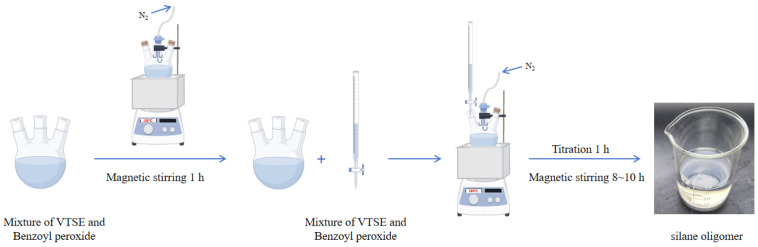
Preparation process diagram of silane oligomer.

**Figure 2:**
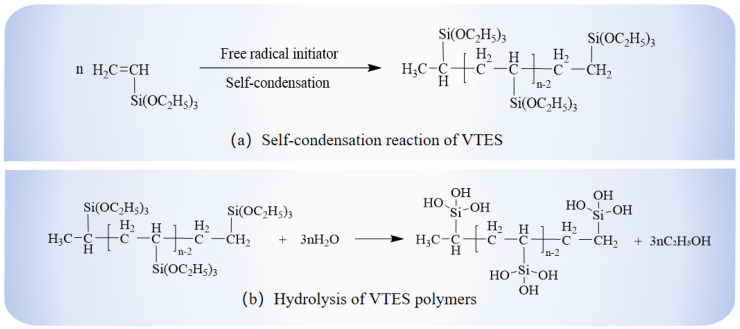
Reaction mechanism of VTES oligomers formation and hydrolysis.

The hydrophobic anti-icing coating materials were synthesized utilizing the free radical polymerization method. Firstly, a predetermined amount of VTES and BPO (1 wt %) was added to a three-neck flask. The mixture was magnetically stirred at 100 °C for 1 h. Subsequently, the remaining VTES monomer and BPO initiator (1 wt %) mixture was placed in a burette. And the mixture in the burette was dropped into a three-necked flask within 1 h. The reaction was maintained under the constant reaction temperature of 100 °C and magnetically stirred for 8 to 10 h. In order to ensure the purity of the reaction environment, the whole reaction process was carried out under a nitrogen gas atmosphere. The final products were silane oligomers with the color of a slightly transparent or pale yellow (Figure 1). Finally, the final products were collected and stored in a sealed indoor environment. The reaction mechanism of VTES oligomer formation and hydrolysis is shown in Figure 2. In order to ensure that the coating covers the substrate surface more evenly, the spraying method was adopted to evenly spray the coating onto the substrate surface in this study [23]. The coating was prepared using spraying technology and cured at room temperature. This spraying method only requires a compressor and a spray gun.

### 2.2. Design of Mixtures

Considering that the asphalt surface layer and cement concrete surface layer are commonly used pavement surface layer forms, therefore, three types of mixtures, AC-13, PAC-13, and PCC, were selected for the comprehensive performance evaluation of the coating.

#### 2.2.1. Asphalt Mixtures

**Figure 3:**
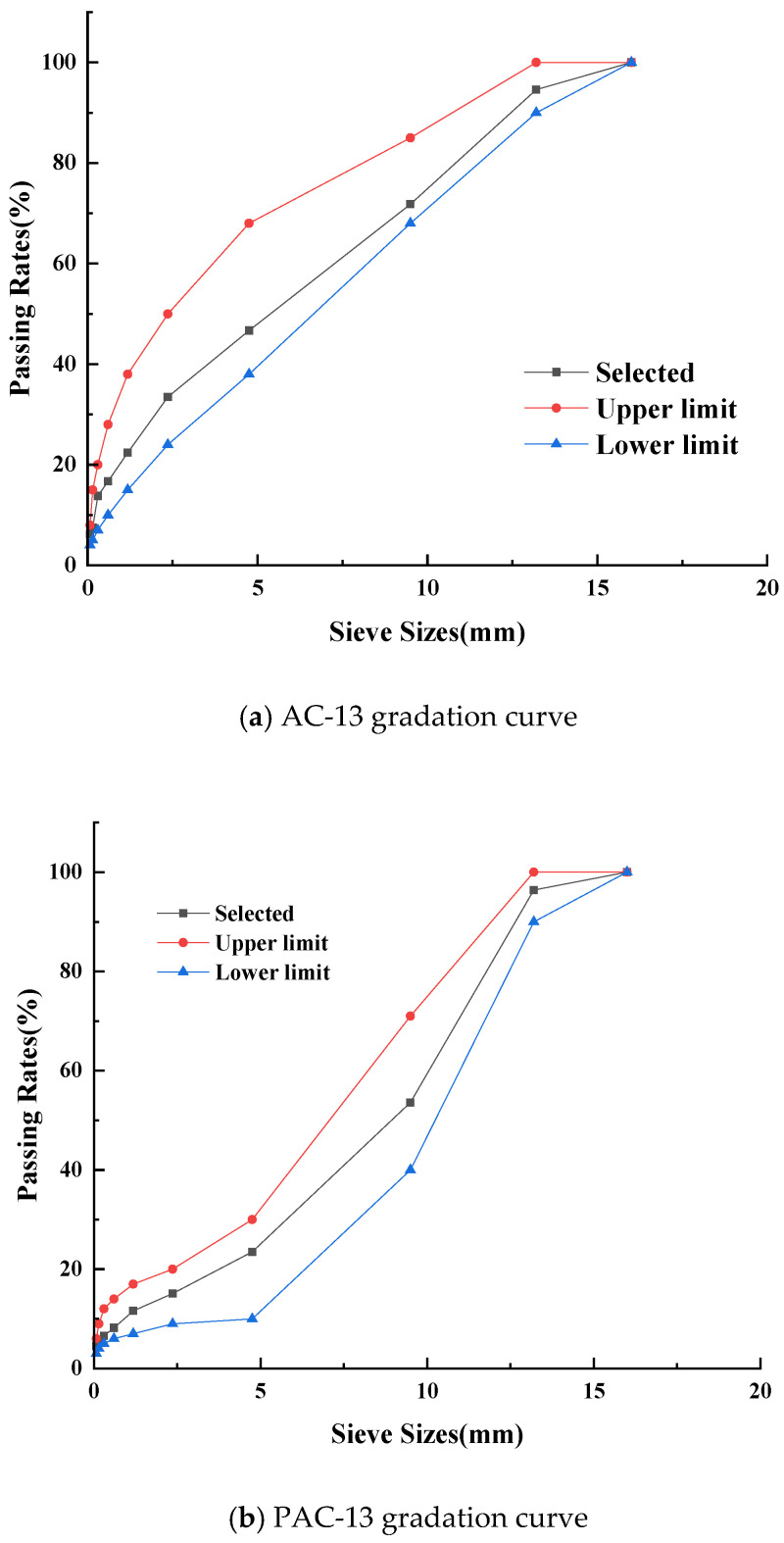
The grading curves of AC-13 and PAC-13.

Considering that both AC-13 and PAC-13 are commonly employed as surface materials for asphalt pavement. Therefore, a comparative study on the surface of AC-13 and PAC-13 was conducted to verify the anti-icing performance of the coating. According to the standard of JTG/T 3350-03-2020 and JTG F40-2004 [24,25], AC-13 and PAC-13 graded asphalt concretes were prepared, with their grading curve shown in Figure 3. For the AC-13 specimens, base asphalt was utilized with an optimal asphalt content of 4.7%, while the PAC-13 specimens employed high-viscosity modified asphalt, with an optimal asphalt content of 4.3%. According to the standard of JTG E20-2011 [26], various asphalt mixtures were prepared.

#### 2.2.2. Portland Cement Concrete Mixtures

The Portland cement concrete (PCC) mixture specimens were prepared in accordance with the standard GB/T 50081-2019 [27]. The Portland cement (PO 42.5) was used in this paper. And coarse aggregates consisted of crushed basalt with particle sizes ranging from 5 to 20 mm, while the natural river sands were used as the fine aggregates. The water–cement ratio was designed as 0.4.

### 2.3. Characterization and Performance Test

The research on the comprehensive performance of hydrophobic anti-icing coatings is very imperfect, and there is no unified standard for test indicators and methods of the hydrophobic anti-icing coating materials for highway pavements. Thus, in this study, from the perspective of the anti-icing performance, durability, and skid resistance performance, the comprehensive performance of hydrophobic coatings was studied through self-designed tests and in combination with existing standard test equipment and technologies.

#### 2.3.1. Contact Angle and Microstructure of Surface Test

To investigate the differences in hydrophobic performance of the hydrophobic anti-icing coating on asphalt and cement concrete substrates, the CAs of coatings and substrates were observed. Additionally, in order to study the relationship between hydrophobic properties and microstructures of coatings, the surface microstructure of coatings was investigated via scanning electron microscopy (SEM, SU8020, Hitachi, Japan), with an accelerating voltage of 5 kV. The samples need to be metalized before the SEM investigation.

For the CA tests, the hydrophobic anti-icing coating was sprayed over the specimen surfaces and was cured until completely cured. Subsequently, a 5 μL droplet of deionized water was deposited onto the coating surface through a microsyringe. Then, the CA was measured through the CA measuring instrument (DSA-100, KRUSS, Germany). Each specimen was tested at three different locations, and the average value was recorded as the CA of the coating. The microstructure of the coating was observed through SEM.

#### 2.3.2. Anti-Icing Performance Test

The morphology of water droplets on pavement surfaces is the visual perception of the hydrophobicity of the coating. Water on the road surface can roll off the road surface, which can significantly reduce the amount and accumulation of ice that forms on the road surface. Therefore, both of icing rate and water droplet morphology were used to evaluate the hydrophobic anti-icing effectiveness of coatings.

A hydrophobic coating material was sprayed over a dry and clean substrate. The area of the substrate was designed as S. When the coating is fully cured, the initial mass of substrate with coating was weighed and designed as m_1_. Then, the coated substrate was placed into the environment with the temperature maintained at −5 °C. After the temperature of the substrate stabilized, about 5 mL of water was dropped vertically on the surface of the substrate from a specific height by the rubber tip dropper. The morphology of the water droplets on the surface was observed. Until the water on the specimen was completely frozen, the weight was recorded again and designated as m_2_. At the same time, a set of uncoated specimens was set up as a comparison. The freezing rate (I) was calculated as follows (Equation (1)).

(1) $$I=\frac{m_{2}-m_{1}}{S}$$

To assess the hydrophobic performance of the coating, 5 mL deionized water droplets from a specific height were deposited on the surface of the coated and uncoated specimens. The enlarged droplet shapes were captured using a digital microscope (W1-B, Shocrex, China) with representative images selected for comparative analysis.

#### 2.3.3. Ice–Pavement Interface Adhesion Strength Test

Reducing the adhesion strength between ice layers and pavement surfaces is crucial for facilitating ice removal and enhancing anti-icing performance. Therefore, the ice–pavement interface adhesion strength serves as a key indicator for evaluating the effectiveness of anti-icing coatings.

**Figure 4:**
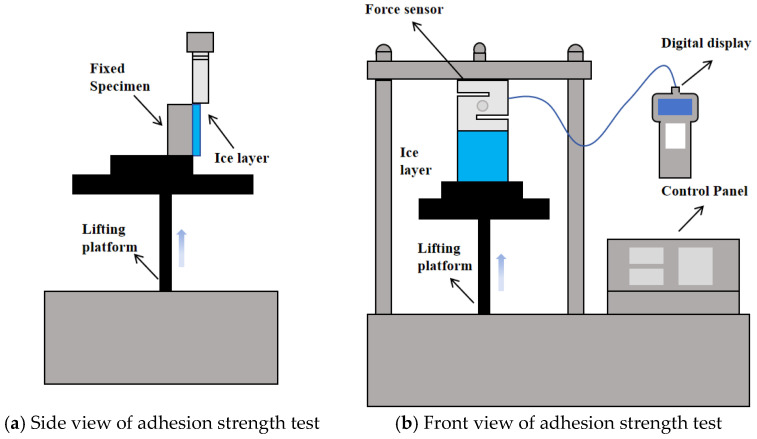
Testing system of ice–pavement interfacial adhesion strength.

In order to study the adhesion strength between ice layers and pavement surfaces, the ice–pavement adhesion strength test has been designed in this paper (Figure 4). After the coating sprayed on the surface of the specimen was completely cured, the specimen was placed in a specific mold, and water was poured on the surface, and then it was placed into the environment with the temperature maintained at −5 °C. The specimen with an ice layer with a thickness of 0.8 cm was formed on the specimen surface. At the same time, a set of uncoated specimens was set up as a comparison.

Subsequently, the ice–pavement interfacial adhesion force was recorded by the adhesion test device. The maximum force was regarded as the ice–pavement interfacial adhesion force, and the corresponding strength was considered the ice–pavement interfacial adhesion strength.

#### 2.3.4. Durability Performance Test

The coating on the road surface will cause wear under the long-term action of the vehicle, so it is necessary to study the durability of the coating. Considering that both the AC-13 and PAC-13 are asphalt mixtures, a uniform abrasion test disc specimen [26] was used for the durability performance test. Moreover, the cement concrete specimens of the abrasion test were prepared as square thin plates. After spraying the hydrophobic coating on the specimen surfaces and fully curing, referring to the standard of JTG E20-2011 [26], a wet wheel abrasion test was conducted to simulate the wear of the coating by the tire. The CA of the specimen before (θ_Before_) and after (θ_After_) the wet wheel abrasion test was measured separately. The reduction rate of the CA (R_θ_) can be calculated by Equation (2). The durability of the coating is judged by R_θ_ of the coating and whether the θ_after_ is greater than 90°.

(2) $$R_{\theta }\left(\%\right)=\frac{\theta_{\mathrm{Before}}-\theta_{\mathrm{After}}}{\theta_{\mathrm{Before}}}\;\times \;100\%$$

#### 2.3.5. Skid Resistance Performance Test

**Figure 5:**
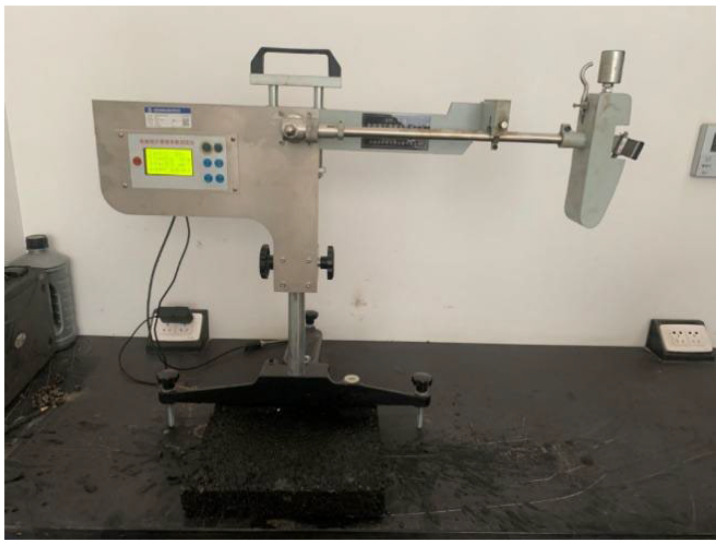
Digital British pendulum test device.

**Figure 6:**
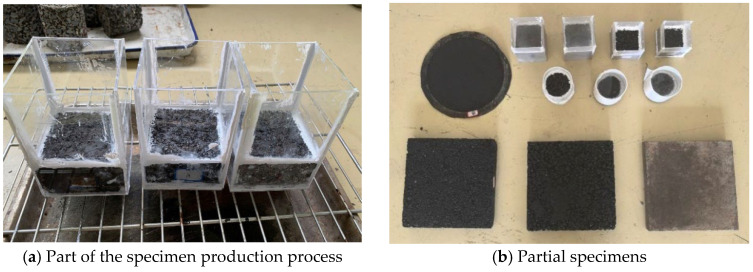
Preparation of specimens for comprehensive performance tests.

The anti-skid performance of the road surface has a significant impact on the safety of road driving. When the anti-skid performance of the road surface is insufficient, vehicles may have safety hazards such as rollover and skidding. Thus, it is necessary to study the skid resistance properties of the coated road surface. Following the JTG 3450-2019 [28], the rutting slab specimens of AC-13, PAC-13, and PCC were prepared to simulate pavement structures. Each specimen was coated with the hydrophobic anti-icing coating and cured completely. Subsequently, the British Pendulum Number (BPN) of specimens was obtained through the BM-V digital pendulum friction tester (Shuyang Highway Instrument Co., Ltd., China) in Figure 5. Comparing the BPN values changes of the specimens before and after coated, the influence of coating material on the skid resistance of different pavement surfaces was researched. The comprehensive performance evaluation methods were listed in Table 1. Some specimens for comprehensive performance tests are shown in Figure 6.

## 3. Results and Discussion

### 3.1. Contact Angle and Microstructure of Surface

**Figure 7:**
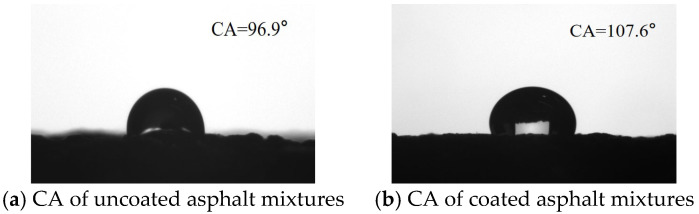
Surface wettability of uncoated and coated asphalt mixtures.

The surface wettability of the substrate can be characterized by the CA. When the CA is less than 90°, the surface exhibits hydrophilic properties. Conversely, when the CA exceeds 90°, the surface displays hydrophobic properties. The CAs of the surface of asphalt mixtures before and after coated are shown in Figure 7. The uncoated asphalt specimen surface demonstrates an average contact angle of 96.9°. While the average CA for the asphalt mixtures after hydrophobic anti-icing coating increased to 107.6°. This indicates that the hydrophobic properties were greatly improved after the surface of the asphalt mixture was coated with hydrophobic coating materials.

**Figure 8:**
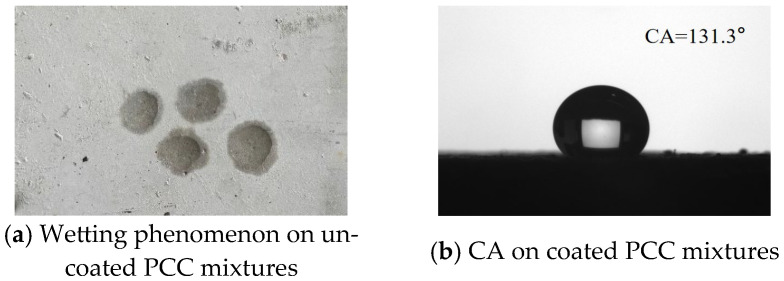
Surface wettability of uncoated and coated PCC mixtures.

It can be seen in Figure 8a that water can infiltrate the PCC surface. This also indicated that the PCC surface exhibits high hydrophilicity and a strong tendency for wetting [7,29,30]. The hydrophobic property of PCC was significantly improved, and the CA for the PCC after hydrophobic anti-icing coating was about 131.3° (Figure 8b). The PCC demonstrated superior hydrophobic performance.

Wenzel wetting model (Equation (4)) describes the effect of surface roughness on wettability [29,31].

(3) $$\frac{\gamma_{SV}\;-\;\gamma_{SL}}{\gamma }=cos\theta_{E}$$

(4) $$cos\theta *=fcos\theta_{E}$$

In Equations (3) and (4), the $\gamma_{\mathrm{SV}}$, $\gamma_{\mathrm{SL}}$, and γ represent the interfacial tensions of solid–vapor, interfacial tensions of solid–liquid, and interfacial tensions of liquid–vapor, respectively. The $\theta^{*}$ represents the CA, while $\theta_{E}$ is the intrinsic static CA. The parameter f is the roughness ratio, which is defined as the ratio of the actual contact area to the projected area. As referenced in the literature, the contact angles determined by Young, Wenzel, or Cassie–Baxter equations correspond to the minimization of the droplet’s free energy. By incorporating the Gibbs free energy formula for the liquid droplet under the constraint of constant volume, we can rigorously derive Young’s equation (Equation (3)). Equation (3) assumes that the solid surface is smooth. When considering the surface roughness of the substrate, the actual contact area between the liquid and the solid is greater than the projected area of the liquid on the horizontal plane, so the apparent contact angle of the Wenzel model is obtained by introducing a parameter f. Therefore, the contact angle is closely related to the interfacial tension and surface roughness.

Application of the hydrophobic anti-icing coating results in the reduced $\gamma_{\mathrm{SV}}$ of the mixtures. Therefore, the θ of mixtures was increased after coated, which results in the increased of $\theta_{w}$ of mixtures and the enhanced hydrophobic performance of mixtures after coated. Furthermore, comparing with asphalt mixtures, PCC mixtures with the rougher microscopic surface led to a higher roughness ratio and greater CA. Thus, PCC mixtures exhibited better hydrophobic properties.

After the hydrophobic anti-icing coating curing on the glass slide, the surface of the coating and cross-sectional morphology were characterized using scanning electron microscopy (SEM).

**Figure 9:**
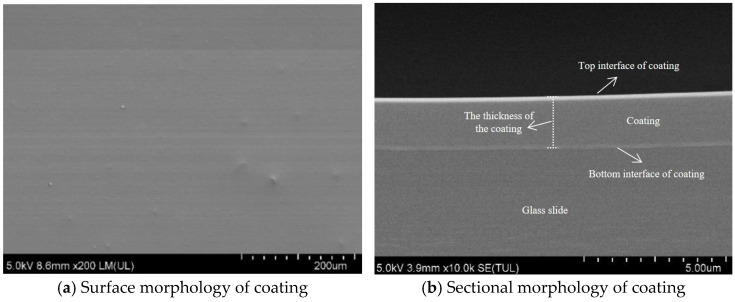
SEM images of the hydrophobic anti-icing coating cured on the glass slide surface.

As shown in Figure 9a, the coating surface cured on the glass slide surface exhibits smooth and uniform microscopic morphology without visible cracks or pores. This indicates that the cross-linking was relatively homogeneous during the curing process of coating materials. It can be seen that the coating materials exhibit excellent flowability and film-forming properties. In Figure 9b, the coating displayed a relatively consistent thickness. The thickness of the coating at different positions was measured, and the average value was regarded as the thickness of the coating (about 1.85 µm). The coating adheres well to the substrate surface. There is no delamination or bubbles inside the coating. That enhances the resistance of the coating to external loads and environmental stress.

### 3.2. Anti-Icing Performance

**Figure 10:**
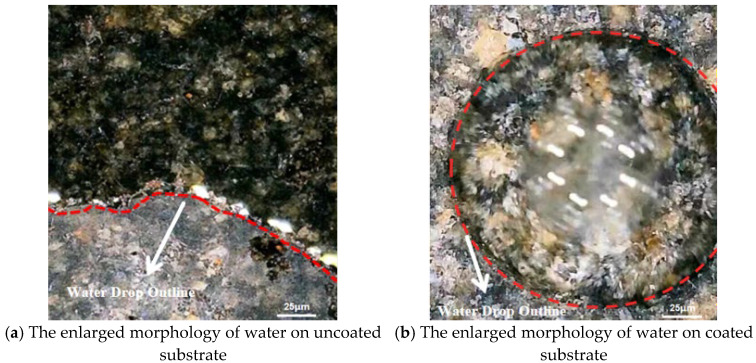

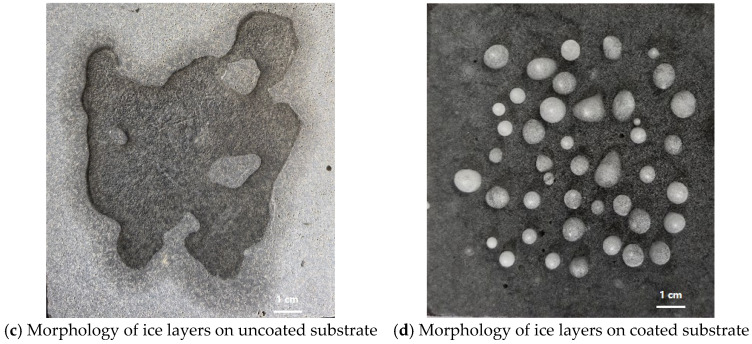
Morphology of water and ice on uncoated and coated substrates.

The morphology of the droplets on the uncoated and coated substrates is shown in Figure 10.

In Figure 10a, the water droplets fall on the uncoated substrate and spread rapidly to all sides, and the uncoated substrate shows strong hydrophilicity [32]. Conversely, Figure 10b shows that the water was distributed in a spherical shape on the coated substrate surface. This results in a significantly smaller contact area between water droplets and the coated substrate surface than that on the uncoated substrate.

The icing rates of the coated substrate and the uncoated substrate were 0.03 g/cm^2^ and 0.04 g/cm^2^, respectively. Compared with the uncoated substrate surface, the icing rate of the coated surface was reduced by approximately 25%. Figure 10c shows that water easily flows on the surface of the uncoated substrate, seeps into the slab, and eventually forms a large area of thin ice. In contrast, water on the coated substrate surface in Figure 10d is scattered on the surface of the substrate, and some water droplets roll off, and finally, the water on the surface gathers to form an irregular hemispherical or irregular convex shape distribution. It can be seen that the hydrophobic anti-icing coating effectively prevents the formation of continuous thin ice layers.

The VTES undergoes polycondensation under the initiation of BPO. The organosilicon oligomers undergo hydrolysis and cross-linking on the surface of the substrate to form a dense polymeric network through dehydration and condensation. The formation of this network structure greatly reduces the surface free energy and weakens the interaction force between the coating and the water or ice [33]. From Equations (3) and (4), it can also be seen that reducing the surface free energy of the substrate resulted in an increase in the CA between water and the coated substrate surface. Thus, water can easily roll off the surface of the substrate, reducing the amount of ice formation on the surface of the material. Furthermore, the interaction between water and low surface energy materials is weak, and it is difficult for water molecules to enter the pores of the road surface through capillary action. This also greatly reduces the infiltration of water into the structure of the pavement, making it easier to remove the ice from the road surface.

**Figure 11:**
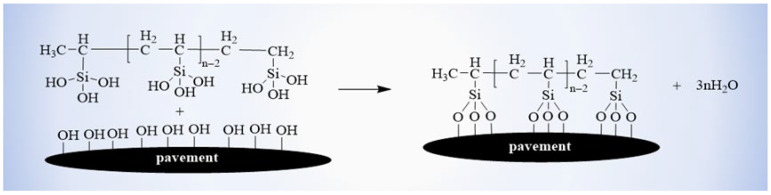
Chemical grafting of hydrolysate of VTES to pavement.

When the coating materials were sprayed on the substrate surface, some reactive groups may also react with the hydroxyl groups (-OH) in the substrates to improve the interfacial bond property between coating and substrate (Figure 11) [33].

### 3.3. Durability Performance

**Figure 12:**
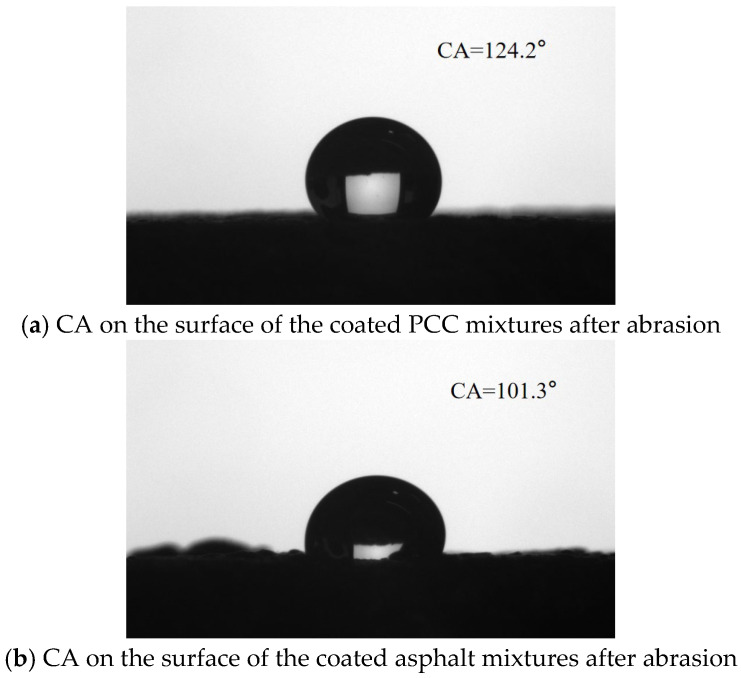
CA on the surface of the coated mixtures after abrasion.

Figure 7 illustrates that the hydrophobic anti-icing coating applied to PCC mixtures reached an initial CA of 131.3°. After undergoing abrasion testing, the CA of the PCC mixture’s surface decreased slightly to approximately 124.2° (Figure 12). Overall, the CA of coating PCC mixtures has decreased by approximately 5%. Similarly, the initial CA of the coated asphalt mixtures was 107.6°, then decreased to 101.3° after abrasion (Figure 12). Overall, the CA-coated asphalt mixtures have decreased by approximately 6%. The CA of coated PCC mixtures was generally greater than that on the surface of coated asphalt mixtures. On the surface of the coated PCC mixtures, the hydrophobic performance decreased slightly after abrasion, but overall, the specimens still maintain the high hydrophobic properties. On the surface of the coated asphalt mixtures, the CA of the surface of coated mixtures was also reduced after abrasion, but the surface of the coated mixtures after abrasion still maintained the hydrophobic characteristics. This demonstrates that the prepared coating has a good potential for practical use.

The durability of hydrophobic anti-icing coatings on the surfaces of PCC mixtures is slightly better than that on asphalt mixtures. This advantage of durability is primarily attributed to the microscopic pore structure of mixtures. The protective mechanism of silane coatings relies on the penetration of its molecules into the capillary pores of concrete, where they undergo hydrolysis and polycondensation reactions with hydroxyl groups (-OH) on the pore walls to form a hydrophobic siloxane network structure according to reference [33,34]. The capillary pores on the concrete surface can accelerate solution penetration [34], and the increasing concrete porosity resulted in the higher permeability coefficients [35]. Therefore, the dense yet fine pore structure of concrete facilitates the penetration of silane coating materials, thereby promoting the formation of an internal hydrophobic film. This network structure formed by chemical bonding can enhance the adhesion of the coating to the PCC mixture’s surface, so that the coating is not easy to fall off during the mechanical wear process, and it also helps prolong the hydrophobic performance of the coating. Whereas the asphalt surface is smoother, the adhesion of the coating to the asphalt mixture’s surface is weaker. As a result, the hydrophobic properties of the coated on the asphalt mixture were much reduced during wear.

### 3.4. Ice–Pavement Interface Adhesion Strength

**Figure 13:**
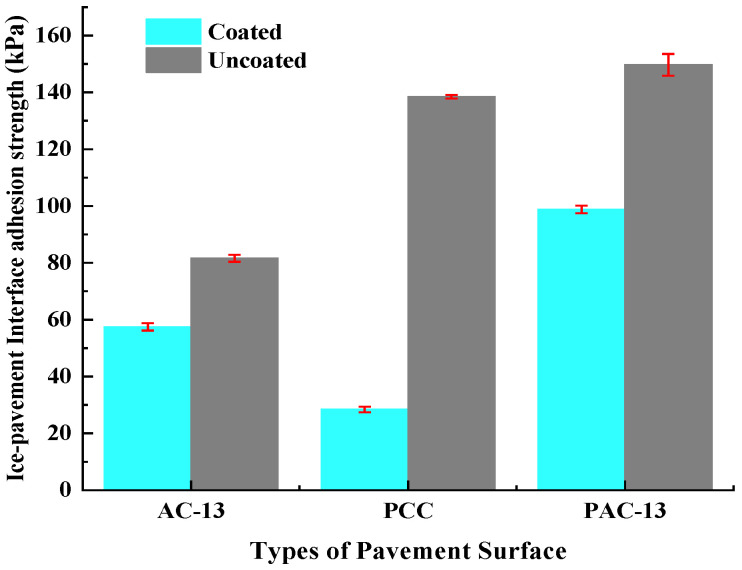
Ice–pavement interface adhesion strength under different types of pavement surface.

Figure 13 and Table 2 illustrate the ice–pavement interface adhesion strength under different types of pavement surfaces. For uncoated pavement surfaces of AC-13, PCC, and PAC-13, the ice–pavement interfacial adhesion strengths were 81.6 kPa, 138.5 kPa, and 149.7 kPa, respectively. For coated AC-13, PCC, and PAC-13 pavement surfaces, the ice–pavement interfacial adhesion strengths were decreased to 57.5 kPa, 28.4 kPa, and 98.8 kPa. Comparing with the uncoated pavement surface of AC-13, PCC, and PAC-13, the ice–pavement interfacial adhesion strengths after coated hydrophobic anti-icing coating were reduced by approximately 30%, 79%, and 34%. This demonstrates that the hydrophobic anti-icing coating significantly reduces the ice–pavement interfacial adhesion strength under the three surface types of pavement surface structures above. Among them, when the coating adheres to the surface layer of the concrete mixture (PCC), the effect of reducing the interfacial adhesion force between ice and the pavement is the most significant.

For uncoated mixtures, the ice–pavement interfacial adhesion force of PAC-13 mixtures was significantly greater than that of AC-13 mixtures. This is due to the large internal voids in PAC-13 mixtures, which make it easy for water to penetrate into the specimen, and ice crystals are formed in situ at low temperatures, which mechanically interlock with aggregates. Therefore, the ice–pavement interface adhesion strength of the PAC-13 mixtures is large. In contrast, AC-13 mixtures with the dense-graded structure inherently resist water penetration, which resulted in ice formation mainly on the surface. Thus, the AC-13 interface adhesion strength was prominent lower than that of PAC-13. For PCC mixtures with a large number of capillary-size pores, water can penetrate into the PCC mixtures and freeze into ice at low temperatures. Thus, the ice–PCC mixture interface adhesion strength is also large.

After spraying the hydrophobic de-icing coating on the road surface, the free energy of the road surface is reduced, the aggregation of hydrogen bonds between the road surface and water molecules is also reduced, and the orderly arrangement between the original road surface and water is dispersed. Thereby, the adhesion between the pavement surface and water or ice molecules is also reduced [36,37]. For coated PCC mixtures, water does not easily penetrate into the capillary-size pores. It resulted in the reducing of the interfacial adhesion force of the PCC. Moreover, according to the Wenzel model, the surface of the PCC mixtures is rougher than that of the asphalt mixtures, which makes the contact angle larger, so the coating also has the best effect on reducing the ice–pavement interfacial adhesion force of the PCC mixtures (Equation (4)).

Furthermore, the ice–pavement bonding model (Equation (5)) based on the quasi-liquid layer theory shows that the interfacial bond strength can be calculated according to the surface CA of the material, the natural temperature, the melting temperature of the quasi-liquid layer, and the surface tension [38,39,40]. The hydrophobic anti-icing coating can significantly reduce the surface energy of the pavement, thereby reducing the surface tension of the surface, and increasing the contact angle of the pavement surface, which ultimately leads to a significant weakening of the adhesion between the ice layer and the pavement surface.

(5) $$\sigma_{\mathrm{ad}\;}=\frac{2\gamma (\cos\theta_{i}+\cos\theta_{s})/b}{a/b-\log(T_{f}-T_{a})}$$

In the equation, $\sigma_{\mathrm{ad}}$ represents the ice–pavement bonding strength (MPa), γ represents the liquid surface tension (MPa), $T_{f}$ represents the melting temperature of ice (°C), $T_{a}$ represents the ambient air temperature (°C), $\theta_{i}$ represents the CA between ice and quasi-liquid layer (°), $\theta_{s}$ represents the CA between quasi-liquid layer and substrate surface (°), and a and b are constants (typically a = 32 nm, b = 21 nm).

**Figure 14:**
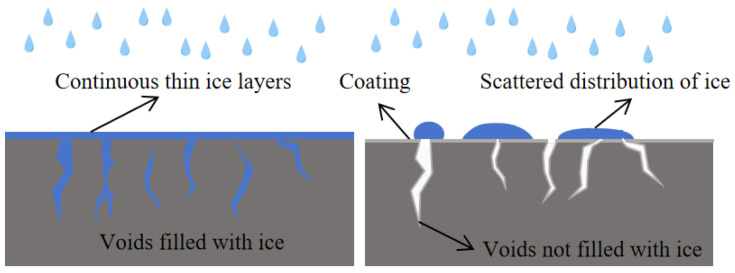
Schematic diagram of the anti-icing mechanism.

During winter rainy and snowy weather, due to the existence of macroscopic structures and fine cracks of the road surface, water is prone to seep into the pores of the structures and cracks, and form “ice whiskers” in the pores, tightly nailed to the road surface. The ice layer formed by rainwater would also cover the surface of the road surface evenly. This also increases the adhesion between the ice layer and the pavement, making it difficult to remove the ice layer. When the road surface is sprayed with hydrophobic materials, the CA of the road surface is significantly increased, which significantly improves the hydrophobic performance of the road surface. It greatly reduces moisture ingress into pores within the pavement. Water on the hydrophobic pavements is distributed as scattered water droplets, which can greatly reduce water infiltrate into the pavement structure. Even if water droplets freeze, it is also easier to remove (Figure 14).

### 3.5. Skid Resistance Performance

**Figure 15:**
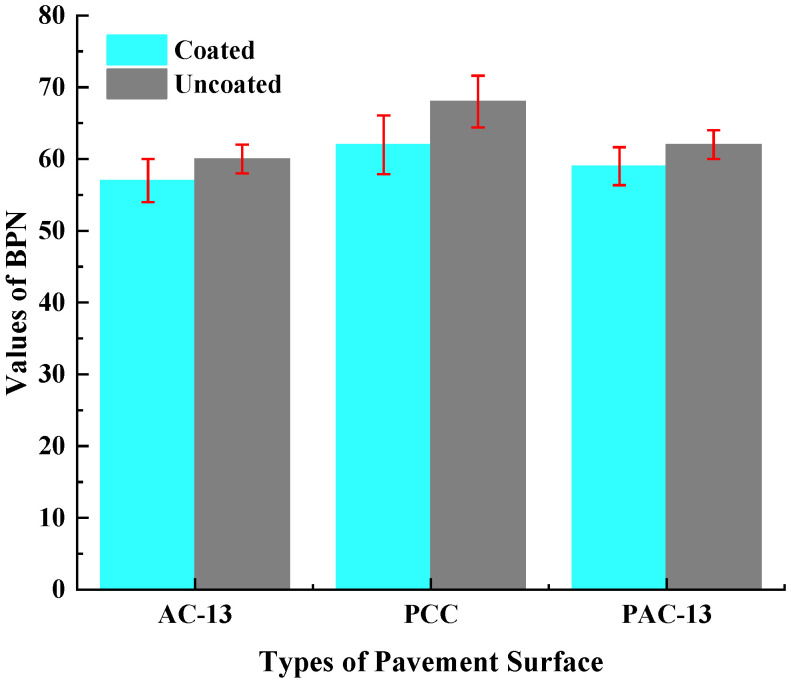
Effects of spray-coated layer on pavement BPN values.

According to Figure 15 and Table 3, the BPN values for AC-13, PCC, and PAC-13 mixtures were measured at 60, 68, and 62, respectively. After spraying the hydrophobic anti-icing coating, the BPN values for AC-13, PCC, and PAC-13 mixtures were measured at 57, 62, and 59, respectively. Compared with uncoated mixtures, these values indicate a maximum reduction of approximately 8.8% for three types of pavements after coated. However, the BPN values of the pavements after hydrophobic coated were still meets the requirements of standard (JT/T 1239-2019 [41]). The spraying of the coating did not significantly change the anti-skid performance of the pavement’s surface.

The coating material with silane oligomers exhibits excellent penetration capabilities. This allows the coating material to penetrate into the microscopic pores of the pavement without changing the roughness structure of the surface. Meanwhile, the coating material was uniformly distributed across both the surface and internal pores of the pavement, leading to a slight reduction in the BPN. However, the thinness of the coating-averaging was approximately 1.85 µm (Figure 9). It exerted minimal influence on the surface texture and structural characteristics of the pavement. Therefore, after spraying coating materials on pavement, the anti-skid performance of the pavement will not be significantly reduction.

## 4. Conclusions

Severe icing on road surfaces significantly impacts traffic safety and efficiency. A de-icing method with the characteristics of high efficiency, environmental friendliness, convenient construction, and low cost has become a focal point in road engineering. In this study, a hydrophobic anti-icing coating for highway pavements was prepared through a free radical polymerization method using VTES as the monomer and BPO as the initiator. The anti-icing performance, durability, and skid resistance performance of the hydrophobic anti-icing coating under the different types of road surface layer structures were evaluated. The conclusions are as follows:

(1) The hydrophobic anti-icing coating demonstrated excellent hydrophobic properties on substrate surfaces. Water does not accumulate easily on the surface of the slate sprayed with anti-icing coating. And it was distributed scattered in a semi-spherical shape. Compared to the uncoated substrate, the hydrophobic anti-icing coating reduced the icing rate by approximately 25%.

(2) Both cement pavements and asphalt pavements after the application of hydrophobic anti-icing coating expressed the hydrophobic properties, and the CAs of these reached 131.3° and 107.6°, respectively. After wet wheel abrasion tests, the coated cement pavements and asphalt pavements still expressed the hydrophobic properties, and the CAs were reduced by 5% and 6% (CAs of 124.2° and 101.3°, respectively). The coating exhibits excellent durability under mechanical wear.

(3) Through designing the ice–pavement interface adhesion strength test, the adhesion strength between ice layers and pavement surfaces has been measured in the paper. Comparing with the uncoated pavements mixtures, for AC-13, PCC, and PAC-13 pavements, the ice–pavement interface adhesion strength after the application of hydrophobic anti-icing coating reduced by 30%, 79%, and 34%, respectively. This means that even if ice formed on the coated surfaces, it can also be more easily removed.

(4) The sprayed of hydrophobic anti-icing coating has little effect on the skid resistance properties of the road surface. The BPN values of AC-13, PCC, and PAC-13 pavements after coated hydrophobic anti-icing coating were 57, 62, and 59, respectively. The spraying of the coating did not significantly change the anti-skid performance of the pavement surface. The skid resistance performance of pavement surfaces coated with the hydrophobic anti-icing coating met the specification requirements.

(5) In this study, the application potential for road anti-icing of hydrophobic coating materials has been confirmed in the laboratory stage. However, the anti-icing effect of hydrophobic coatings in practical engineering is affected by multiple factors, such as vehicle load and the surrounding environment. In the future, it is necessary to apply the hydrophobic coatings to the actual roads to research the comprehensive performance and service life in actual road environments.

## Figures and Tables

**Table 1:** Comprehensive performance evaluation methods.

Tests	Index	Design of Tests
Contact Angle and Microstructure of Surface Test	CA; Microstructure of Surface	Self-designed test
Anti-Icing Performance Test	Freezing Rate	
lce-Pavement Interface Adhesion Strength Test	Interface Adhesion Strength	
Durability Performance Test	Reduction Rate of CA	Self-designed test and JTG E20-2011 [26]
Skid Resistance Test	BPN	JTG 3450-2019 [28]

**Table 2:** Ice–pavement interface adhesion strength under different types of pavement surface.

Types of Pavement Surface	Uncoated (kPa)	Coated (kPa)	Reduction Rate of Interfacial Adhesion Strength (%)
AC-13	81.6	57.5	30
PCC	138.5	28.4	79
PAC-13	149.7	98.8	34

**Table 3:** Effects of spray-coated layer on pavement BPN values.

Types of Pavement Surface	Uncoated	Coated	BPN Reduction Rate (%)
AC-13	60	57	5.0
PCC	68	62	8.8
PAC-13	62	59	4.8

## Data Availability

The original contributions presented in this study are included in the article. Further inquiries can be directed to the corresponding author.
